# Integrated Prevention at Work: Protocol for a Concept Analysis

**DOI:** 10.2196/29869

**Published:** 2021-06-17

**Authors:** Alexandra Lecours, Marie-Ève Major, Claude Vincent, Valérie Lederer, Marie-Ève Lamontagne

**Affiliations:** 1 Département d'ergothérapie, Université du Québec à Trois-Rivières Trois-Rivières, QC Canada; 2 Center for Interdisciplinary Research in Rehabilitation and Social Integration Québec, QC Canada; 3 Faculté des sciences de l’activité physique, Université de Sherbrooke Sherbrooke, QC Canada; 4 Centre de recherche interdisciplinaire sur le bien-être, la santé, la société et l’environnement Université du Québec à Montréal Montreal, QC Canada; 5 Département de réadaptation, Université Laval Québec, QC Canada; 6 Département des relations industrielles, Université du Québec en Outaouais Gatineau, QC Canada

**Keywords:** integrated prevention at work, concept analysis, occupational injury, meta-narrative review, qualitative research, focus groups

## Abstract

**Background:**

Integrated prevention at work promises to eliminate the boundaries between primary, secondary, and tertiary prevention actions taken by stakeholders in the world of work. It is receiving increasing attention from the scientific community because of its concerted and harmonized approach, which promotes employment access, return, and healthy long-term continuation. Although promising, integrated prevention is not yet well-defined, which makes it difficult to operationalize.

**Objective:**

This manuscript exposes the protocol of a study aiming to conceptualize integrated prevention at work on the basis of scientific and experiential knowledge.

**Methods:**

Using a concept analysis research design, data collection has been planned in 2 parts. A meta-narrative literature review will first be conducted to document how integrated prevention has been defined in the literature. Then, phone interviews will be conducted with key informers (ie, managers, workers, ergonomists, occupational therapists, psychologists, physiotherapists, union and insurance representatives) to document their viewpoints and understanding of integrated prevention at work. Qualitative data gathered during these 2 parts of research will be analyzed using template analysis, which allows data from literature and empirical collection to be analyzed simultaneously. The analysis will bring out the points of convergence, divergence, and complementarity between the information gleaned from literature and key informers’ experiences to arrive at a conceptualization of integrated prevention at work by identifying its uses, attributes, antecedents, and consequences. As a final step, validation and interpretation with a TRIAGE (Technique for Research of Information by Animation of a Group of Experts) group will be carried out in collaboration with the key informers to identify the tools for the implementation of integrated prevention at work and promote workers’ health and safety.

**Results:**

This study is expected to offer a contemporary conceptualization of integrated prevention at work that clearly lays out the variables of this concept and elicits the viewpoints of the different stakeholders.

**Conclusions:**

This study will contribute to the advancement of knowledge about the professional injury prevention continuum. The clear identification of the uses, attributes, antecedents, and consequences of integrated prevention at work will offer concrete tools to stakeholders to implement innovative and promising approaches to integrated prevention at work.

**International Registered Report Identifier (IRRID):**

PRR1-10.2196/29869

## Introduction

Work is an activity practiced by millions of people and valued across cultures and societies [1]. It is estimated that more than 50% of the global population participates in the labor market [2], and the number of people employed is on the increase in industrialized countries. In Canada, the number of workers rose from 15.8 to 20.2 million between 2000 and 2019, which represents a jump of nearly 28% [3]. Identified as a determinant of health [4,5], work can have positive effects on an individual’s health, well-being, and quality of life. For example, in addition to providing financial security, work can be a source of social recognition, protection against the decline of certain faculties, and social contacts [6-8]. However, work is not without risk, and can also have negative effects on people. Occupational injuries, whether related to a workplace accident or physical or mental illness, may diminish an individual’s work participation and quality of life. Not only do occupational injuries affect workers and their families, but they also have repercussions on work organizations, particularly by increasing absenteeism [9] or decreasing performance [10]. The social impacts of occupational injuries are also considerable, with an estimated cost of €680 billion (US $828 billion) in the European Union [11] or US $171 billion in the United States [12], only for the year 2019. The contemporary COVID-19 pandemic situation that has afflicted the planet since the end of 2019 adds to the daily risks that threaten workers. Stress of contracting the virus, accessing appropriate protective equipment, constantly changing policies and procedures, modifications in work tasks, obligation to telework, or uncertainties in the work schedule are examples of the new risks afflicting workers [13-15]. In doing so, constant and innovative efforts for the prevention of occupational injuries are required to promote healthy work participation.

Various primary (ie, to prevent their appearance), secondary (ie, to reduce their duration), and tertiary (ie, to prevent prolonged disability) prevention practices coexist to manage occupational injuries [2]. These practices respond to different needs and are implemented independently by many stakeholders at different levels, such as the work environment (eg, managers, unions or workers), health care and social services system (eg, ergonomists and rehabilitation professionals), or insurance systems (eg, rehabilitation advisors) [16]. This compartmentalized prevention model makes communication and consultation between stakeholders difficult [16,17]. In fact, it has been noted that few bridges exist in practice between the various levels of prevention, and there are few links between stakeholders where the management of prevention practices is concerned [17]. The benefits of eliminating the boundaries between actions in primary, secondary, and tertiary prevention by various stakeholders to adopt an approach of integrated prevention at work have been suggested [18]. Integrated prevention is garnering increasing interest from the scientific community because it would help support injured workers’ employment access, return, and healthy long-term continuation [19]. Actions traditionally associated with each of the 3 levels of prevention would then not be mutually exclusive and would gain from being integrated to increase benefits across the prevention continuum [18]. In addition to generating significant economic advantages in terms of effectiveness and resource allocation [18], integrated prevention would allow stakeholders to work synergistically [20], promoting a less fragmented prevention approach [18]. Although considered a promising avenue, integrated prevention is still an emerging concept and is not well-defined [17]. This gap in current knowledge makes it difficult to operationalize the concept, thereby impeding its implementation by stakeholders. Moreover, the concept was first defined in ergonomics, and described mainly in terms of workplace prevention interventions [17]. It would be required to ensure that the concept is formed from a perspective that includes the whole prevention continuum (eg, including also the rehabilitation level) so that all stakeholders share a common understanding of the concept.

The aim of this study is to conceptualize integrated prevention at work on the basis of scientific and experiential knowledge. The study will be guided by 3 research questions: (1) How is integrated prevention defined in the literature? (2) What are stakeholders’ viewpoints on integrated prevention at work? (3) What are the points of convergence, divergence, and complementarity between the information gleaned from literature and stakeholders’ experiences?

## Methods

### Design

A concept analysis research design [21] will be used to structure the study. This design goes beyond the linguistic analysis of a concept, leading to a theoretical construction [21]. It enables the integration of both scientific and experiential knowledge to define the variables to operationalize the concept, which are uses, attributes, antecedents, and consequences [21]. Concept analysis has been used in other occupational rehabilitation studies to better understand certain concepts such as mental workload [22] or preventive behavior at work [23].

### Procedure and Participants

**Figure 1:**
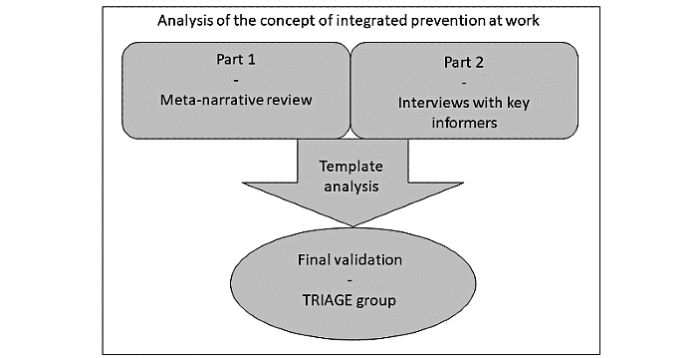
Study design. TRIAGE: Technique for Research of Information by Animation of a Group of Experts.

Data collection consists of 2 parts conducted in parallel, as shown in Figure 1.

#### Part 1

##### Meta-narrative Review

A *meta-narrative literature review* will first be carried out to collect information from literature [24]. This documentary research technique facilitates the interpretation of term meanings [25], which makes it compatible with the concept analysis research design. Furthermore, meta-narrative reviews propose a systematic method that offers the flexibility required to include diverse types of documents (eg, scientific articles and gray literature) [24]. To increase scientific rigor of the protocol, the Preferred Reporting Items for Systematic Review and Meta-analysis Protocols (PRISMA-P) recommendations [26] will be included, although they are not traditionally applied to meta-narrative literature reviews.

##### Documentary Search

To answer the question, “How is integrated prevention at work conceptualized?,” a research strategy (ie, keywords and Boolean operators, eg, *integrated prevention AND work,* and databases, eg, *MEDLINE*) will be validated by a consultant librarian to ensure that all the fields of relevant literature are covered (eg, ergonomics, prevention, rehabilitation, management). The quality of the strategy will be evaluated according to Peer Review of Electronic Search Strategies (PRESS EBC) Elements criteria [27,28]. The reference lists of the selected documents will also be examined manually to ensure literature saturation. Moreover, the gray literature will be explored to include theses, professional papers, institutional documents, and textbooks. To do so, a search in Google with the same keywords as those used for the scientific databases will be performed. The results generated in the first 4 pages will be explored [29].

##### Selection of Manuscripts

Two members of the research team will conduct the documentary search according to the following inclusion criteria: (1) manuscripts concerning work and (2) manuscripts addressing the concept of integrated prevention (ie, documents proposing a definition of integrated prevention or a description of uses, attributes, antecedents, or consequences). For feasibility reasons, only documents written in French or English will be selected, and no limit regarding publication dates will be imposed.

##### Selection Study

The selected documents will be integrated into the Covidence reference management software. After the elimination of duplicates, 2 members of the research team will check for the relevance of all documents based on the title, abstract, and keywords. If ambiguity is present, the document will be read entirely to determine its inclusion in the study. Regular peer-debriefing meetings among the researchers will take place to rule on the inclusion or rejection of the documents [30]. This regular communication among researchers will heighten their reflexivity and guard against the undue influence of any one’s perspective.

##### Data Extraction

Finally, the information will be extracted from the selected manuscripts by 2 members of the research team using an extraction grid constructed according to concept analysis–specific variables [22]. The extraction grid will include considerable descriptive information about the manuscripts (eg, authors and country), methodological information (eg, participants and study design), and results (eg, uses, attributes, antecedents, and consequences of integrated prevention at work). The use of the grid will be piloted during the data extraction of 3 manuscripts and the interrater reliability will be tested. To ensure the quality of the meta-narrative review, Realist And Meta-narrative Evidence Syntheses: Evolving Standards (RAMESES) [25] criteria will be respected.

#### Part 2

##### Interviews With Key Informers

*Phone interviews will be conducted among key informers* drawn from stakeholders in the occupational injury prevention continuum (ie, managers, workers, ergonomists, occupational therapists, psychologists, physiotherapists, union and insurance representatives). Key informers must have at least two years of work experience, be involved in 1 or more phases of the continuum of occupational injury prevention, and be fluent in French. They will be recruited through purposive sampling method and selected using a maximum variation strategy [31] to obtain diversity in terms of sex, age, workplaces, kind of implication toward prevention, and years of experience. The number of participants will be determined as the study progresses when saturation and redundancy are reached in the data gathered. Given the specificity of the subject of the study, the initial number of participants estimated is between 12 and 20 [32,33]. Indeed, some authors suggested that, for qualitative studies aiming to describe the experiences of people sharing a similar reality, interviews with a dozen participants are generally sufficient to achieve saturation of the results [32]. The final number of participants will be adjusted during the study and recruitment will stop when the interviews reveal a redundancy in the sense of the ideas reported by the participants [32]. Interviews of approximately 60 minutes in length will be conducted to elicit key informers’ viewpoints and understanding of integrated prevention at work. Questions about definitions, uses, attributes, antecedents, and consequences of the concept will be asked according to a pretested interview guide. Interview recordings will be fully transcribed into verbatim. Sociodemographic data of participants will also be collected.

### Analysis

Descriptive statistics will be done on sociodemographic data of the participants. Qualitative data extracted from articles (part 1) and interview transcription (part 2) will be analyzed using template analysis, a type of thematic analysis strategy [34,35]. This strategy is compatible with concept analysis and allows data from literature and empirical collection to be analyzed simultaneously.

A 5-step analysis will follow: (1) Several readings of the entire corpus will be done to obtain an overall sense. (2) The initial coding will start, and descriptive codes will be assigned to meaning units (single ideas) found in the data corpus. The aim of the study will be kept in focus to ensure the relevance of the proposed coding, as the codes are intended to help define the uses, attributes, antecedents, and consequences of the concept of integrated prevention at work. (3) The next step will consist of delineating categories and themes. The codes (micro level) will be classified into categories (meso level) and broader themes (macro level). Based on the concept analysis design, 4 a priori themes (ie, 1: uses, 2: attributes, 3: antecedents, and 4: consequences) will be used. (4) Thereafter, a general structure will be generated, and this step will enable the researchers to establish links between the selected codes, categories, and themes. (5) The raw data will be applied to the general structure multiple times to fine-tune the analytical process. This analytical process will be undertaken by 2 members of the research team, and interrater agreement will be monitored periodically. The QDA Miner [28] software program will be used to support this process. The analysis will triangulate results from the 2 parts of the study to answer the research questions, allowing to (1) highlight how literature defines integrated prevention at work; (2) present key informers’ viewpoints; and (3) identify points of convergence, divergence, and complementarity between the data extracted from literature and the information collected from key informers. A conceptualization of integrated prevention at work with the identification of variables of interest (ie, uses, attributes, antecedents, and consequences) will emerge from the analysis. In addition to the identification of the variables that make up the concept, a graphic representation illustrating it and highlighting the links between these variables will be generated. Periodical meetings of research team members during the process of analysis will lead to the creation of successive versions of the understanding of the concept of integrated prevention at work until everyone agrees that the analysis performed represents the data as closely as possible.

### Final Validation

A virtual discussion group between the research team and some of the key informers encountered during part B will be held to validate and interpret the results. A synthesis of results and the proposed conceptualization of integrated prevention at work will be sent to key informers a month before the meeting [36]. These documents will be discussed during a TRIAGE (Technique for Research of Information by Animation of a Group of Experts) [37,38] group meeting with participants (n=8) who took part in the study. The TRIAGE method allows discussions among participants to be structured so as to arrive at a consensus on specific ideas while facilitating the emergence of new ideas [37]. This method was shown to be economical and rigorous to reach a consensus on emerging subjects [37]. Various indicators (eg, applicability, relevance, clarity) will be documented.

The data will be interpreted in situ, which is consistent with the TRIAGE method [37,38]. This last consultation step will allow obtaining a common and shared conceptualization of integrated prevention. Intervention ideas to encourage the implementation of an integrated prevention approach by stakeholders will also emerge from this last step.

## Results

*From a research perspective*, this study will contribute to the advancement of knowledge about the occupational injury prevention continuum. This study is expected to offer a contemporary conceptualization of integrated prevention at work that clearly lays out the variables of this concept and elicits the viewpoints of the different stakeholders. Such a definition could allow researchers interested in this subject to refer to a common definition, thus facilitating the comparison of research results. In a next step, a formal validation study to obtain a broader consensus from the scientific and practicing communities on the proposed conceptualization will be carried out. The research team will also be able to develop a concept measurement tool and an intervention model that can be used by stakeholders.

*From a clinical and organizational perspective*, the clear identification of variables of integrated prevention at work (ie, uses, attributes, antecedents, and consequences) will provide concrete tools to stakeholders for the implementation of innovative and promising approaches.

*From a societal perspective*, this study will offer avenues of intervention to innovate regarding prevention of occupational injuries. These avenues will be all the more useful because they will have been developed taking into account the emerging risks encountered by workers in connection with the COVID-19 pandemic. It is hoped that the future projects that will be carried out following this study will help reduce the societal costs linked to occupational injuries.

## Discussion

This study uses a design involving data collection in 2 parts to be able to access a large pool of knowledge on integrated prevention at work from literature and data collection. In addition, the participation of different key informers from the world of work (eg, workers, employers, rehabilitation professionals, insurers) ensures that the results will be relevant to their reality, and optimizes the chances that the ideas for action that come out of the study are adopted in practice. The methodology of the study is described in detail to ensure scientific rigor and replicability. Nevertheless, as this study is conducted in Canada, the transferability of the results to other contexts cannot be guaranteed.

This article presents a research protocol to conceptualize integrated prevention at work on the basis of scientific and experiential knowledge. The results of this study will lay the ground for a contemporary conceptualization of occupational injury prevention in which the contribution of stakeholders is clearly explained. It is hoped that the results of this study will pave the way for further studies and interventions to ensure healthy work participation for all workers.

## Appendix

Multimedia Appendix 1 Peer-review report by IRRST-REPAR (Quebec, Canada).

## Abbreviations

PRESS: Peer Review of Electronic Search Strategies
PRISMA-P: Preferred Reporting Items for Systematic Review and Meta-analysis Protocols
RAMESES: Realist And Meta-narrative Evidence Syntheses: Evolving Standards
TRIAGE: Technique for Research of Information by Animation of a Group of Experts
