# Supplementary material for: Integrated Prevention at Work: Protocol for a Concept Analysis
Source: JMIR Res Protoc. 2021 Jun 17;10(6):e29869. doi: 10.2196/29869 (PMC8277315; doi:10.2196/29869)
Supplement: Multimedia Appendix 1 [file resprot_v10i6e29869_app1.pdf]

4.1 IRSST-REPAR Volet 3 - Évaluation scientifique

79pts

1. Qualité scientifique

18/20pts

A. Nécessité et originalité de l'étude

○ 0 ○ 1 ○ 2 ○ 3 ◉ 4 ○ 5

B. Clarté des objectifs de recherche

○ 0 ○ 1 ○ 2 ○ 3 ○ 4 ◉ 5

C. Clarté, pertinence et rigueur de la démarche scientifique

○ 0 ○ 1 ○ 2 ○ 3 ○ 4 ◉ 5

D. Importance des résultats escomptés

○ 0 ○ 1 ○ 2 ○ 3 ◉ 4 ○ 5

Commentaire

Le projet de recherche est convaincant et intéressant dans son ensemble. Les différentes étapes de réalisations sont bien décrites et choisies pour répondre aux objectifs déterminés.
La problématique est bien décrite. Par ailleurs, les auteurs ne mentionnent pas ce qui a été réellement fait autour du concept de prévention intégrée, si elle est actuellement utilisée, si elle reste un concept théorique et si elle a été utilisée ailleurs qu'au Québec. Est-ce une concept tiré de l'ergonomie et très utilisé qu'on souhaite maintenant appliquer plus largement auprès de l'ensemble des acteurs ? Est-ce uniquement un concept scientifique ou est-ce tout de même un concept utilisé en clinique ? Ces éléments pour moi sont manquants.
Les objectifs de l'étude sont très clairs et bien décrits. On comprend que le volet 1 et le volet 2 sont associés aux deux premiers objectifs et que l'objectif 3 se fait via les groupes de discussions, mais cela aurait pu être clarifié.
Méthode bien décrite et claire.

2. Faisabilité

29/35pts

A. Réalisme de l'échéancier

○ 0 ○ 1 ○ 2 ○ 3 ○ 4 ○ 5 ○ 6 ○ 7 ◉ 8 ○ 9 ○ 10

B. Réalisme du budget demandé

○ 0 ○ 1 ○ 2 ○ 3 ○ 4 ◉ 5

C. Potentiel de pérennité de l'initiative

○ 0 ○ 2 ○ 4 ○ 6 ○ 8 ○ 10 ○ 12 ○ 14 ◉ 16 ○ 18 ○ 20

Commentaire

Dans l'ensemble la faisabilité du projet me semble très bien.
Je me demande pas ailleurs s'il est réaliste de compléter les entrevues de recherche après 10 mois vu le besoin d'obtenir l'approbation éthique pour le projet et les délais que cela peut engendrer, mais cela reste faisable.
La contribution d'une professionnelle de recherche et d'une étudiante de maîtrise contribuent à la faisabilité vu le travail qu'elles pourront accomplir ! Le budget me semble donc réaliste puisqu'il est tourné vers l'implication de ces deux personnes.
Je pense que le projet peut certainement mener à une pérrennité de l'initiative.
Un élément qui a aussi soulevé des questions est l'utilisation du terme "transfert des connaissances intégré". Je

comprends que sur l'échéancier, c'est pour cette raison que le TC a été mis à travers l'ensemble du projet, mais ce que je vois dans les méthodes de transfert sont des méthodes "a postériori".

3. Qualité et complémentarité de l'équipe

32/45pts

A. Complémentarité des demandeurs (expertises, méthodologies ou domaines de recherche)

○ 0 ○ 3 ○ 6 ○ 9 ○ 12 ○ 15

B. Productivité scientifique et rayonnement selon le stade d'évolution de la carrière

○ 0 ○ 1 ○ 2 ○ 3 ○ 4 ○ 5 ○ 6 ○ 7 ○ 8 ○ 9 ○ 10

C. Importance du rôle et de la contribution de chacun des membres de l'équipe

○ 0 ○ 1 ○ 2 ○ 3 ○ 4 ○ 5 ○ 6 ○ 7 ○ 8 ○ 9 ○ 10

D. Contexte favorable au maillage entre chercheurs et partenaires sociaux ou à la migration de chercheurs en provenance d'autres disciplines vers la réadaptation au travail

○ 0 ○ 1 ○ 2 ○ 3 ○ 4 ○ 5 ○ 6 ○ 7 ○ 8 ○ 9 ○ 10

Commentaire général à propos de cette évaluation ou de la demande

Je pense que ce projet de recherche a beaucoup de potentiel afin de mieux établir les bases d'un concept qui pourrait être utile pour supporter les travailleurs et travailleurs blessé(e)s. Par ailleurs, j'aurais aimé mieux comprendre comment le concept de prévention intégré peut vraiment être utilisé dans le continuum de réadaptation et comment ce dernier peut avoir un impact concret sur le bien-être des travailleurs(-euses). Cela n'est toujours pas clair pour moi, le bénéfice de cette approche à plus long terme.

Aussi, il est mentionné que la chercheuse possède des contact avec un comité de travailleurs(-euses) de l'Estrie et qu'ils pourront contribuer au recrutement, mais comment ces personnes pourraient contribuer plus directement au projet pour que la vision des travailleurs(-euses) soient toujours placées au centre des préoccupations dans l'étude.

Un autre élément à réfléchir est la discipline des chercheuses impliquées. 3 sont des ergos (+ une étudiante et professionnelle de recherche), une est ergonome et l'autre. Comment s'assurer que la vision du projet ne sera pas professionnalisante et qu'elle gardera cette vision plus large (gestion, travailleurs, assureurs). Il aurait été bien de diversifier l'équipe en ce sens.

4.1 IRSST-REPAR Volet 3 - Évaluation scientifique

80pts

1. Qualité scientifique

15/20pts

A. Nécessité et originalité de l'étude

○ 0 ○ 1 ○ 2 ○ 3 ○ 4 ○ 5

B. Clarté des objectifs de recherche

○ 0 ○ 1 ○ 2 ○ 3 ○ 4 ○ 5

C. Clarté, pertinence et rigueur de la démarche scientifique

○ 0 ○ 1 ○ 2 ○ 3 ○ 4 ○ 5

D. Importance des résultats escomptés

○ 0 ○ 1 ○ 2 ○ 3 ○ 4 ○ 5

Commentaire

For the key informants, where applicable, consider that they should have direct experience with workers and not just a minimum of 2 years work experience.
Further details on the development of the interview guide or a draft would have been helpful.

2. Faisabilité

28/35pts

A. Réalisme de l'échéancier

○ 0 ○ 1 ○ 2 ○ 3 ○ 4 ○ 5 ○ 6 ○ 7 ○ 8 ○ 9 ○ 10

B. Réalisme du budget demandé

○ 0 ○ 1 ○ 2 ○ 3 ○ 4 ○ 5

C. Potentiel de pérennité de l'initiative

○ 0 ○ 2 ○ 4 ○ 6 ○ 8 ○ 10 ○ 12 ○ 14 ○ 16 ○ 18 ○ 20

3. Qualité et complémentarité de l'équipe

37/45pts

A. Complémentarité des demandeurs (expertises, méthodologies ou domaines de recherche)

○ 0 ○ 3 ○ 6 ○ 9 ○ 12 ○ 15

B. Productivité scientifique et rayonnement selon le stade d'évolution de la carrière

○ 0 ○ 1 ○ 2 ○ 3 ○ 4 ○ 5 ○ 6 ○ 7 ○ 8 ○ 9 ○ 10

C. Importance du rôle et de la contribution de chacun des membres de l'équipe

○ 0 ○ 1 ○ 2 ○ 3 ○ 4 ○ 5 ○ 6 ○ 7 ○ 8 ○ 9 ○ 10

D. Contexte favorable au maillage entre chercheurs et partenaires sociaux ou à la migration de chercheurs en provenance d'autres disciplines vers la réadaptation au travail

○ 0 ○ 1 ○ 2 ○ 3 ○ 4 ○ 5 ○ 6 ○ 7 ○ 8 ○ 9 ○ 10

Commentaire général à propos de cette évaluation ou de la demande

4.1 IRSST-REPAR Volet 3 - Évaluation scientifique

83pts

1. Qualité scientifique

15/20pts

A. Nécessité et originalité de l'étude

○ 0 ○ 1 ○ 2 ○ 3 ○ 4 ○ 5

B. Clarté des objectifs de recherche

○ 0 ○ 1 ○ 2 ○ 3 ○ 4 ○ 5

C. Clarté, pertinence et rigueur de la démarche scientifique

○ 0 ○ 1 ○ 2 ○ 3 ○ 4 ○ 5

D. Importance des résultats escomptés

○ 0 ○ 1 ○ 2 ○ 3 ○ 4 ○ 5

Commentaire

1. Pas de page d'intro et de résumé dans cette demande? Deuxième demande au concours?
2. Le cadre théorique et le contexte de la demande sont clairs. Des exemples pour illustrer les concepts auraient été utiles.
3. Projet très ancré dans les milieux pratiques +
4. Guide d'entrevue et nature des questions inconnues.

Retombées encore vagues et surtout à plus long terme pour l'équipe.

2. Faisabilité

28/35pts

A. Réalisme de l'échéancier

○ 0 ○ 1 ○ 2 ○ 3 ○ 4 ○ 5 ○ 6 ○ 7 ○ 8 ○ 9 ○ 10

B. Réalisme du budget demandé

○ 0 ○ 1 ○ 2 ○ 3 ○ 4 ○ 5

C. Potentiel de pérennité de l'initiative

○ 0 ○ 2 ○ 4 ○ 6 ○ 8 ○ 10 ○ 12 ○ 14 ○ 16 ○ 18 ○ 20

Commentaire

Le calendrier semble réaliste considérant l'intégration de l'équipe dans le milieu et la participation d'acteurs clés.
Mesures adaptées au contexte de la pandémie.
Plus difficile d'envisager les impacts à long terme et la pérennité du projet.
Budget réaliste avec volet formation.

3. Qualité et complémentarité de l'équipe

40/45pts

A. Complémentarité des demandeurs (expertises, méthodologies ou domaines de recherche)

○ 0 ○ 3 ○ 6 ○ 9 ○ 12 ○ 15

B. Productivité scientifique et rayonnement selon le stade d’évolution de la carrière

○ 0 ○ 1 ○ 2 ○ 3 ○ 4 ○ 5 ○ 6 ○ 7 ○ 8 ○ 9 ○ 10

C. Importance du rôle et de la contribution de chacun des membres de l’équipe

○ 0 ○ 1 ○ 2 ○ 3 ○ 4 ○ 5 ○ 6 ○ 7 ○ 8 ○ 9 ○ 10

D. Contexte favorable au maillage entre chercheurs et partenaires sociaux ou à la migration de chercheurs en provenance d’autres disciplines vers la réadaptation au travail

○ 0 ○ 1 ○ 2 ○ 3 ○ 4 ○ 5 ○ 6 ○ 7 ○ 8 ○ 9 ○ 10

Commentaire

Équipe complémentaire avec expériences variées et niveaux de rayonnement différents. Certains chercheurs semblent surtout impliqués dans la première phase du projet, leur rôle par la suite est moins clair.
Projet très ancré dans les milieux dans les phases 2-3 ce qui laisse présager un retour éventuel et un bon potentiel de transfert des connaissances vers ces milieux.

Commentaire général à propos de cette évaluation ou de la demande
